# Induction Heating Optimization for Efficient Self-Healing in Asphalt Concrete

**DOI:** 10.3390/ma17225602

**Published:** 2024-11-16

**Authors:** Marina Penalva-Salinas, David Llopis-Castelló, Carlos Alonso-Troyano, Alfredo García

**Affiliations:** Highway Engineering Research Group, Universitat Politècnica de València, Camino de Vera s/n, 46022 Valencia, Spain; mpensal@upv.edu.es (M.P.-S.); caraltro@upv.es (C.A.-T.); agarciag@tra.upv.es (A.G.)

**Keywords:** self-healing, asphalt mixture, induction heating, ferromagnetic additive, steel slag, sustainable material, energy efficiency

## Abstract

In this study, the practical application of self-healing asphalt mixtures incorporating steel wool fibers and induction heating was investigated, expanding upon previous research that primarily assessed the self-healing properties rather than optimizing the heating process. Specifically, the aim was to enhance the induction heating methodology for a semi-dense asphalt concrete mixture (AC 16 Surf 35/50 S). In this research, the induction heating parameters were refined to improve the self-healing capabilities, focusing on the following three key aspects: (i) energy consumption, (ii) heating rate, and (iii) heating homogeneity. The findings reveal that the current intensity, the percentage of ferromagnetic additives, and coil shape are critical for achieving optimal heating conditions. Higher current intensity and additive percentage correlate with improved heating speed and reduced energy consumption. Additionally, variations in coil shape significantly influence the heating uniformity. Although asphalt mixtures with steel slag coarse aggregates exhibit slightly higher specific heat, this aggregate type is preferable for sustainability, as it allows for the recycling of industrial waste. The optimized mixtures can rapidly reach high temperatures, facilitating effective crack repair. This innovation offers a durable, environmentally friendly, and cost-effective solution for road maintenance, thereby enhancing the longevity and performance of asphalt pavements.

## 1. Introduction

Asphalt mixtures are crucial in the construction of roads and infrastructure due to their excellent mechanical properties and durability. However, over time, these materials tend to fatigue, leading to surface cracks, which can negatively affect performance and result in high maintenance costs. Fortunately, bitumen’s viscoelastic properties allow it to flow under certain conditions and close these cracks, a phenomenon known as the self-healing process [1]. Self-healing technologies are expected to extend material service life, reduce maintenance, and lower raw material consumption, greenhouse gas emissions, and the environmental impact of construction on climate change [2].

As a viscoelastic material, asphalt’s viscosity decreases significantly at high temperatures, making it temperature-sensitive. However, relying on solar radiation for crack healing is impractical due to its uncontrollable nature. Therefore, implementing a regulated heat supply system can facilitate timely and effective crack repair in asphalt [3,4].

To raise the temperature of asphalt mixtures, various heating methods have been explored, including microwave, infrared, and induction heating [4,5,6]. Among these, microwave and induction heating are the most commonly used. While microwave heating is more efficient in terms of heating rate, it tends to produce less uniform heat distribution compared to induction heating [1,7,8,9]. Additionally, microwave heating is less practical for road applications [3,5,6].

Induction heating operates based on Faraday’s law, where electromagnetic currents induce magnetic flux in conductive materials. This flux generates eddy currents that produce heat through the joule effect. Biot–Savart’s law further explains how magnetic fields are influenced by current. Alternating currents cause fluctuations in the magnetic field and the induced currents, generating heat. Consequently, current intensity is a critical factor; increasing it directly accelerates the heating process.

The addition of ferromagnetic materials, such as steel wool fibers, steel shavings, and ferrite particles, has been instrumental in enabling induction heating in asphalt mixtures. Liu et al. [10,11] studied the use of steel wool fibers, finding that longer fibers with smaller diameters improved the mixtures’ electrical conductivity. Similarly, Menozzi et al. [12] and Bueno et al. [13] investigated the use of molten steel particles to repair microcracks, while Zhu et al. [7] utilized ferrite powder in stone mastic asphalt (SMA) to assess microwave heating. More recently, Lizárraga et al. [14] used electric arc furnace slag as a ferromagnetic material, demonstrating significant energy savings in the heating process. Among the various ferromagnetic materials available, steel wool fiber stands out as the most efficient. It is advisable to use thick, short fibers to minimize the risk of clumping or caking, ensuring a uniform distribution of the additive within the mixture.

In addition to adding ferromagnetic materials to enable induction heating in asphalt mixtures, it is also possible to replace portions of natural aggregates with steel slag aggregates, a byproduct of the steel industry. These aggregates have shown effectiveness in improving microwave heating efficiency and reducing the life-cycle costs of asphalt mixtures [15,16,17]. However, it remains to be seen whether these benefits extend to induction heating.

Induction heating is also a key component of the ‘combined healing system’, which enhances the overall crack healing efficiency in asphalt mixtures by synergistically activating both capsule-based rejuvenators and induction heating [9]. This system promotes accelerated rejuvenator diffusion and improved induction healing, leading to longer-lasting pavement.

Despite extensive research on ferromagnetic materials and their effects on self-healing efficiency, there has been a notable lack of focus on optimizing the heating parameters involved in induction heating. Variables such as heating duration, intensity, and the material’s thermal properties have not been thoroughly investigated. Vila-Cortavitarte et al. [15,17] highlighted the need for further exploration of these factors, suggesting that optimizing these parameters could significantly enhance the self-healing performance of bituminous mixtures. This optimization remains a critical gap in the literature on self-repairing asphalt pavements [2].

### 1.1. Induction Fundamentals

Using the joule effect formulas, the power (*P*) required to heat an asphalt mixture can be determined based on the amount ×of mixture (*m*), the desired temperature increase (∆*T*), the material’s heat capacity (*C*), and the heating duration (*t*), as shown in Equation (1) [18]:

(1) $$P=\frac{C\times m\times ∆T}{t}$$

The specific heat capacity (*C*) of a material reflects its ability to store internal energy as heat, representing the energy required to increase the material’s temperature by one unit [19]. Hence, if the material remains constant, its specific heat capacity should also remain constant [20].

Regarding heat distribution, it is important to note that when a conductive material is heated by induction, both the current density and material temperature decrease exponentially with depth. If heating is proportional to the current density (*J*), the temperature distribution (*T*) at different depths can be expressed as in Equation (2) [21]:

(2) Tx=Tsup×e−xδ

where the penetration depth (δ) depends on the angular velocity (*ω*), magnetic permeability (*μ*), and electrical conductivity (*σ*), as shown in Equation (3):

(3) $$\delta =\sqrt{\frac{2}{\omega \times \sigma \times ϻ}}$$

Since the angular velocity (*ω*) is directly related to the frequency (*f*), reducing the frequency lowers the angular velocity, which increases the penetration depth and results in more homogeneous heating [22].

Another important parameter in induction heating is the coil’s shape or geometry. It is crucial to recognize that the principles of heat distribution through induction cannot be directly applied to self-healing asphalt mixtures [23]. In these mixtures, only the added conductive fibers act as conductive elements, with heating primarily occurring at the top surface. However, a similar temperature distribution is expected, with maximum temperatures at the coil’s perimeter and an exponential decrease toward the center.

### 1.2. Objectives and Contribution

The previous research reveals a significant gap in understanding how parameters like current intensity, frequency, coil shape, and asphalt mixture composition impact the effectiveness of induction heating.

Thus, the main objective of this study is to optimize the parameters of induction heating to achieve the most homogeneous and rapid heating possible. To do so, diverse self-healing asphalt concrete (SHAC) mixtures are defined with different types of coarse aggregates and ferromagnetic additives. The influence of parameters such as current intensity, frequency, and coil shape on induction heating is then studied. The aim of this research is to contribute to a significant advance in the implementation of self-healing asphalt mixtures for large-scale infrastructure projects, enhancing their sustainability and durability.

## 2. Materials and Methods

The methodological framework for this study consists of a series of experimental steps designed to evaluate the self-healing potential of the asphalt mixture. First, an asphalt mixture was selected based on its suitability for self-healing applications. Next, a ferromagnetic additive, specifically steel wool fibers, was chosen, and its content was determined based on preliminary tests and manufacturing constraints. Specimens of the asphalt mixture with different fiber contents were then prepared for testing. The induction heating characterization followed, which included several key components, as follows: (i) selection of a suitable induction machine; (ii) analysis of energy consumption and power requirements for heating, taking into account the type of aggregates and the steel wool fiber content; (iii) assessment of the heating rate as a function of aggregate type, fiber content, and induction parameters; and (iv) evaluation of heating homogeneity, which considered variables such as induction frequency and coil shape. These steps collectively provide a comprehensive approach to assessing the feasibility of induction heating for self-healing in asphalt mixtures.

### 2.1. Materials

A semi-dense asphalt concrete mixture, AC16 surf 35/50 S, was selected for this study. The following aggregates were used to design the asphalt mixture: (i) limestone filler, (ii) limestone fine aggregate, (iii) steel slag coarse aggregate, and (iv) porphyry coarse aggregate. The particle size distributions of these materials are detailed in Table 1. Additionally, virgin bitumen 35/50 pen was included. These aggregates and bitumen are commonly used for surface layers in road construction and rehabilitation, accommodating different traffic types and climates.

The selection of the ferromagnetic additive type and its content is supported by the findings from the literature review. Thick, short steel wool fibers are recommended, specifically type 4# steel wool fibers (ø = 0.11–0.17 mm; l = 10 mm; ρ = 7.85 g/cm³). The content of steel wool fibers in the asphalt mixtures was constrained by manufacturing limitations. To further refine this selection, the fiber content was increased in 2% increments in asphalt mixtures of types SHAC S and SHAC P. During this process, it was observed that mixtures with fiber contents equal to or exceeding 6% by volume of bitumen exhibited issues related to homogeneity, which compromised the structural integrity of the material. Consequently, fiber contents of 2% and 4% by volume of bitumen were selected as the optimal levels for this study, balancing performance requirements with manufacturing capabilities.

**Figure 1:**
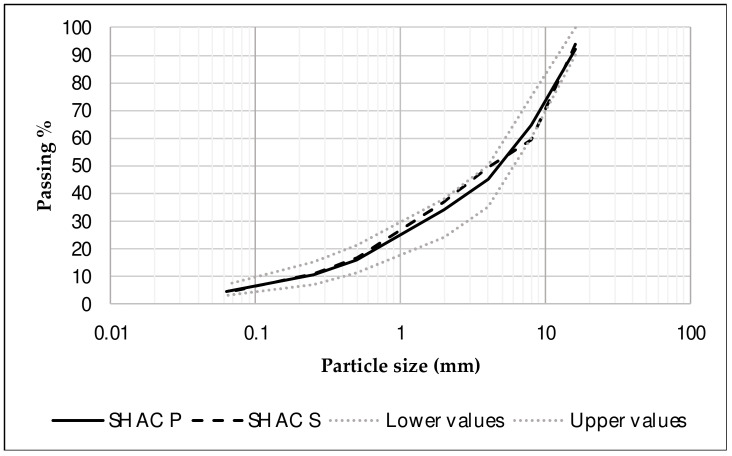
Particle size distribution.

Based on these materials and according to granulometric spindles specified in the Spanish road specifications (PG-3) [25] for an asphalt mixture AC16 surf S (see Table 1 and Figure 1), the following self-healing asphalt concrete (SHAC) mixtures were formulated, as shown in Table 2:

- SHAC S2: composed of limestone filler, limestone fine aggregate, steel slag coarse aggregate, and 2% of steel wool fibers by volume of bitumen.
- SHAC S4: composed of limestone filler, limestone fine aggregate, steel slag coarse aggregate, and 4% of steel wool fibers by volume of bitumen.
- SHAC P2: composed of limestone filler, limestone fine aggregate, porphyry coarse aggregate, and 2% of steel wool fibers by volume of bitumen.
- SHAC P4: composed of limestone filler, limestone fine aggregate, porphyry coarse aggregate, and 4% of steel wool fibers by volume of bitumen.

**Table 2:** Dosage of self-healing asphalt mixtures, in % of total mass.

Type of Asphalt Mixture	Limestone Filler	Limestone Fine Aggregate 0/4	Porphyry Coarse Aggregate 4/11	Porphyry Coarse Aggregate 12/18	Steel Slag Coarse Aggregate 4/11	Steel Slag Coarse Aggregate 10/20	Bitumen	Steel Wool Fibers
SHAC S2	4.76%	42.36%	-	-	29.99%	18.09%	4.17%	0.63%
SHAC S4	4.73%	42.09%	-	-	29.80%	17.97%	4.15%	1.26%
SHAC P2	5.16%	37.58%	32.61%	19.38%	-	-	4.57%	0.69%
SHAC P4	5.13%	37.32%	32.38%	19.25%	-	-	4.54%	1.38%

### 2.2. Preparation of Specimens

The four asphalt mixtures were produced following the Spanish road specifications (PG-3) [25]. Three cylindrical specimens (ø = 101.6 ± 0.1 mm; h = 63.5 ± 2.5 mm) were prepared for each mixture type (12 specimens in total). These specimens were compacted using a Marshall hammer, following the procedure outlined in UNE-EN 12697-30 [26] (75 blows per side), to determine the bulk density of each mixture, as shown in Table 3.

Using the bulk densities, eight prismatic specimens (410 × 260 × 50 mm) were compacted using a roller compactor according to UNE-EN 12697-33 [27] (two specimens per asphalt mixture). These prismatic specimens were used for induction heating characterization. It is important to note that the steel wool fibers were added to the asphalt mixture through a dry process, where they were mixed with the aggregates before adding the bitumen.

### 2.3. Induction Heating Characterization

After preparing the specimens, the induction heating process was characterized using three distinct approaches. First, the power required to heat each asphalt mixture was measured to evaluate energy consumption. Second, the surface heating rate was analyzed by varying the coarse aggregate type, fiber content, current intensity, and frequency. Finally, the influence of coil shape, frequency, and coarse aggregate type on the heating homogeneity of the asphalt mixtures was assessed. This comprehensive approach allows for a thorough understanding of the factors influencing the effectiveness of induction heating in self-healing asphalt mixtures.

**Figure 2:**
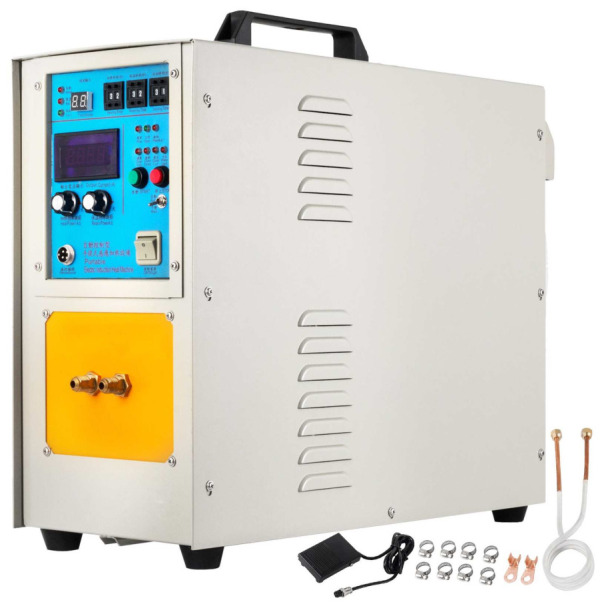
Induction heater.

The induction heater used for this study is shown in Figure 2. It has a power output of 15 kW, with an adjustable current intensity ranging from 200 to 600 A and a frequency range of 30 to 100 kHz.

Before heating, the specimens were conditioned in a refrigerator to reach a temperature of 25 °C. They were held at this temperature for at least two hours to ensure consistent starting conditions for all samples.

#### 2.3.1. Energy Consumption and Power Required for Heating

To optimize energy consumption, the required power for each type of asphalt mixture to achieve a specific temperature increase within a set time frame was compared. The specific heat capacity of each asphalt mixture was determined following ASTM E1952 [28].

First, the heat capacity (*C*) of the calorimeter was determined by mixing cold and hot water and then calculating the equilibrium temperature, as shown in Equation (4).

(4) C=m1×CH2O×(t2−t)t−t1−m1×CH2O(JK)

where *m*_1_ is the mass of cold water; *t*_1_ is the temperature of the cold water (25 °C); m_2_ is the mass of hot water; *t*_2_ is the temperature of the hot water (80 °C); *t* is the equilibrium temperature; and *C_H2O_* is the specific heat of the water.

Next, the specific heat (*C_me_*) of each asphalt mixture was calculated using Equation (5). For that purpose, a sample of asphalt mixture was put into the calorimeter and then stirred, identifying the equilibrium temperature, as follows:

(5) Cme=(m1×CH2O+C)×(t−t1)m2×(t2−t)(Jkg×K)

where *m*_1_ is the mass of water; *t*_1_ is the temperature of the water; *m*_2_ is the mass of asphalt mixture; *t*_2_ is the temperature of the asphalt mixture; *t* is the equilibrium temperature; *C* is the heat capacity of the calorimeter; and *C_H2O_* is the specific heat of the water.

#### 2.3.2. Heating Rate

The influence of the coarse aggregate type (steel slag vs. porphyry), ferromagnetic additive content (2% vs. 4%), current intensity (200 A, 400 A, and 600 A), and frequency (30 kHz, 65 kHz, and 100 kHz) on the heating rate was studied. For each combination of current intensity and frequency (nine combinations in total), the surface temperature of each asphalt mixture was measured. A circular coil with a diameter of 9 cm and three turns was used, with a constant gap (5 mm) between the specimens and the coil.

**Figure 3:**
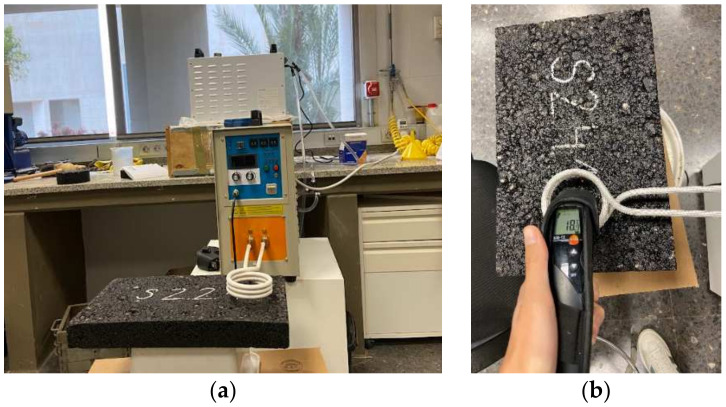
Heating rate characterization: (**a**) heating process and (**b**) temperature measurement.

Each prismatic specimen was heated for 10 min using the induction heater, unless the surface temperature reached 100 °C earlier. The surface temperature was recorded every 30 s using a Testo 830-T2 laser thermometer (see Figure 3).

The combinations of current intensity (I) and frequency (F) are denoted as I1F1, I2F1, I3F1, I1F2, I2F2, I3F2, I1F3, I2F3, and I3F3, where 1, 2, and 3 represent the minimum, medium, and maximum values for the current intensity and frequency, respectively.

#### 2.3.3. Homogeneity of Heating

In the self-healing phenomenon, it is important not only to identify how quickly a certain temperature is reached but also how evenly this temperature is distributed, i.e., the homogeneity of the heating process. To assess the influence of heat transfer from both the base layer and the surrounding air, the temperature gradients between the surface and base of the specimens were analyzed. This approach allowed for an assessment of the heating homogeneity, which is crucial to avoid an excessive surface temperature that could damage the bitumen and to ensure that the internal temperatures are sufficient to facilitate bitumen flow through cracks.

**Figure 4:**
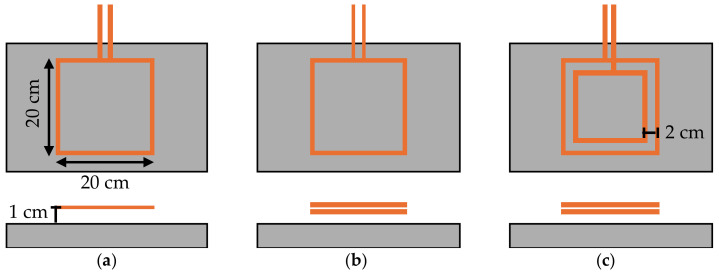
Coil shapes used to characterize heating homogeneity: (**a**) single-turn, (**b**) double-turn, and (**c**) two-turn centered.

To evaluate the heating homogeneity, the surface and depth temperature gradients were measured, varying the induction frequency and coil shape. Three different coil designs were handmade and tested, as follows: (i) single-turn, (ii) double-turn, and (iii) centered two-turn coil (see Figure 4). The surface gradient was measured as the difference between the maximum and minimum temperatures reached on the specimen’s surface, using a FLIR E40 thermal camera. Meanwhile, the depth gradient was calculated as the difference between the surface temperature and the base temperature.

To achieve optimal heating distribution, the spacing between coil turns was empirically set at approximately 2 cm. This decision was informed by observations indicating that temperature peaked at the perimeter of each coil turn and decreased toward the center. By positioning a second coil turn between the center and perimeter of the first turn, heating could be intensified in the cooler regions, resulting in a more uniform temperature distribution. For vertical spacing between the coil and the mixture surface, a distance of 1 cm was maintained. This gap ensures that the coil remains close enough to achieve effective heating while accommodating surface irregularities typical of road materials.

## 3. Results

### 3.1. Power Required for Heating

**Figure 5:**
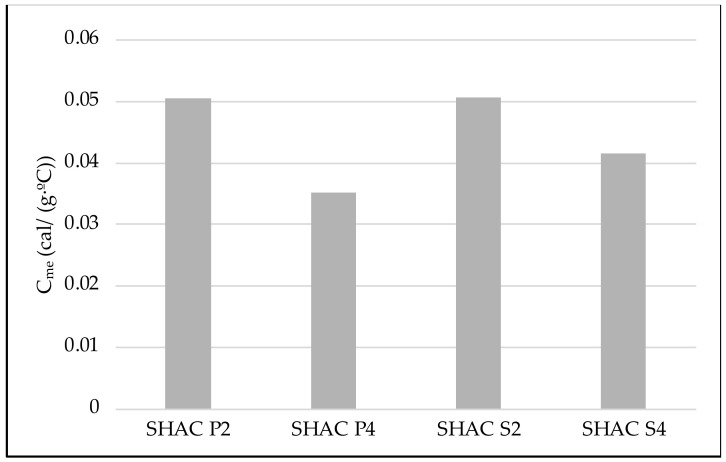
Specific heat values of each sample.

Figure 5 shows the specific heat values of each type of asphalt mixture according to ASTM E1952. As expected, the asphalt mixtures with a higher fiber content require less energy to heat up, resulting in lower specific heat values.

When comparing steel slag and porphyry coarse aggregates, the results are quite similar for a 2% steel wool fiber content. However, with a 4% content of ferromagnetic additives, the asphalt mixture with porphyry coarse aggregate has the lowest specific heat, indicating a higher heating efficiency compared to the asphalt mixture with steel slag coarse aggregate.

### 3.2. Influence of Aggregate Nature and Content of Steel Wool Fibers

**Figure 6:**
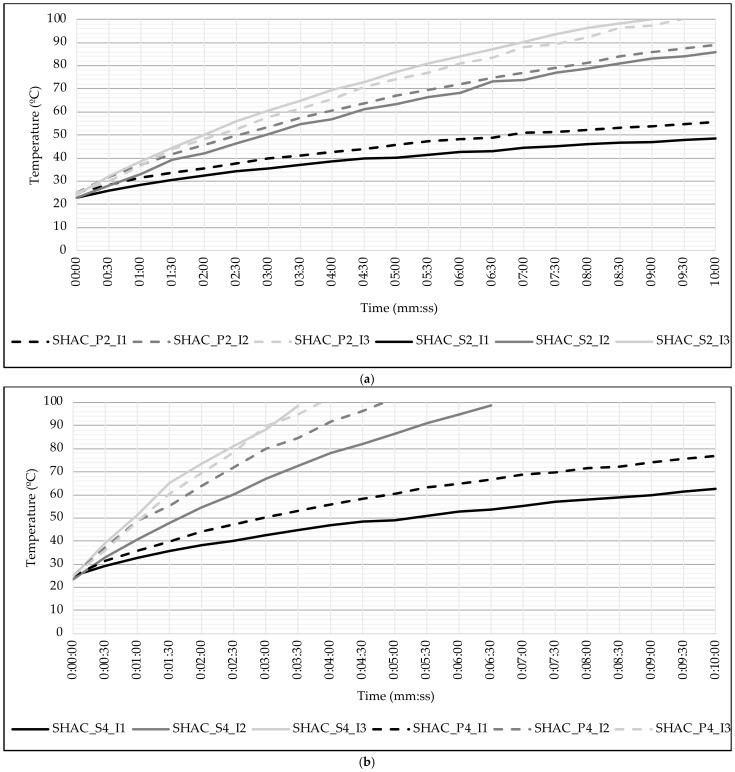
Influence of type of coarse aggregate: (**a**) asphalt mixtures with 2% of steel wool fibers and (**b**) asphalt mixtures with 4% of steel wool fibers.

To analyze the impact of the coarse aggregate type on the heating process, we evaluated the temperature reached by the different asphalt mixtures over time at various current intensities (see Figure 6). The results shown are for the minimum level of induction frequency of (30 kHz), as this parameter had a negligible influence on surface temperature.

As aligned with the findings regarding the calorific values of the asphalt mixtures, porphyry aggregate heats up faster than steel slag aggregate at current levels 1 and 2, regardless of steel wool fiber content. This difference is particularly pronounced with 4% fiber content (see Figure 6b), where the porphyry aggregate reaches approximately 15 °C higher after 10 min of heating at current level 1 and reaches 100 °C more than one minute earlier at level 2. However, when the maximum current level (3) is applied, the temperatures reached by both aggregates tend to converge. In this case, asphalt mixtures with 2% and 4% steel wool fibers reach 100 °C in under 9.5 min and 4 min, respectively.

**Figure 7:**
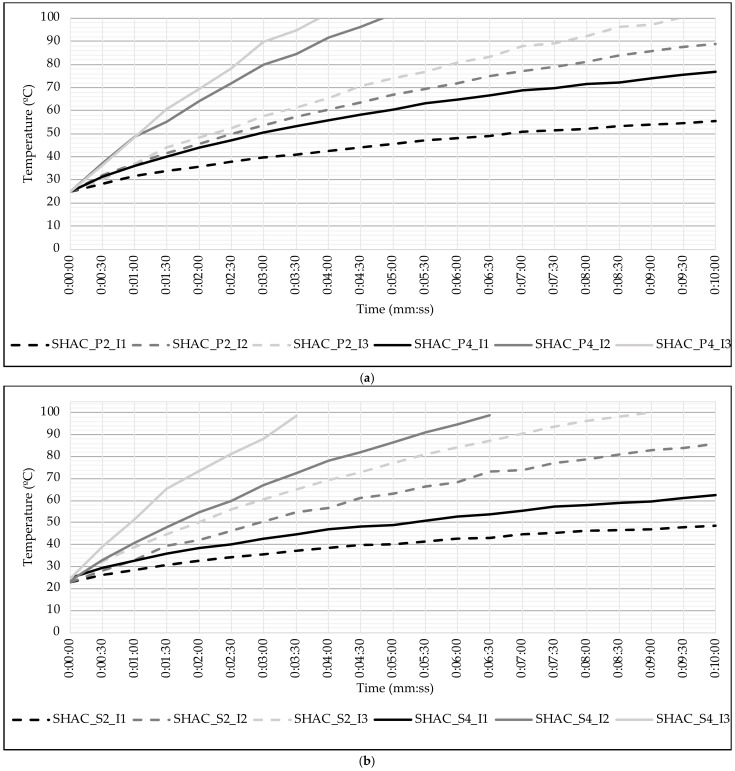
Influence of the content of steel wool fibers: (**a**) asphalt mixtures with porphyry coarse aggregate and (**b**) asphalt mixtures with steel slag coarse aggregate.

Figure 7 shows the heating process for each asphalt mixture, grouping the mixtures by coarse aggregate type. Dashed lines represent the mixtures with 2% steel wool fibers, while solid lines represent the mixtures with 4% fiber content.

For both aggregate types, the heating rate increases with the content of ferromagnetic additives. Asphalt mixtures with 2% steel wool fibers only reached 100 °C at the maximum current level, whereas mixtures with 4% content achieved this temperature even at medium current levels.

Moreover, the difference in heating rates between 2% and 4% fiber content becomes more significant at higher current intensities. The difference in temperature increase between the two fiber contents is smaller at current intensity 1, but becomes more substantial at intensity levels 2 and 3.

Thus, it can be concluded that both fiber content and current intensity are key factors in the induction heating of asphalt mixtures.

### 3.3. Influence of Frequency and Coil Shape

One of the key phenomena in induction heating is the “skin effect”, where alternating current (AC) tends to concentrate near the surface of a conductive material rather than penetrating uniformly. This occurs due to the generation of eddy currents, which create opposing magnetic fields that limit current flow to the material’s outer layers. The depth at which current density significantly decreases is called the “skin depth”, and it depends on the material’s conductivity, magnetic permeability, and induction frequency. In theory, higher frequencies or more conductive materials result in a shallower skin depth, making induction heating highly efficient for surface heating.

In this section of this study, the focus is on analyzing the influence of induction frequency on both surface and depth temperature gradients. For this purpose, different coil designs were manufactured and tested (see Figure 4).

**Figure 8:**
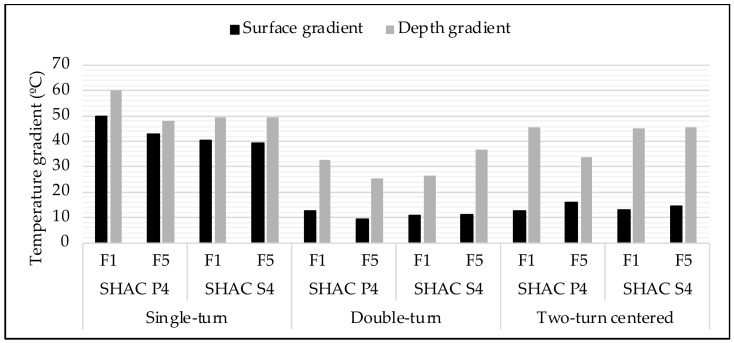
Homogeneity of heating.

Figure 8 compares the surface (in black) and depth (in grey) temperature gradients for different coarse aggregate types, frequency levels, and coil shapes. Only asphalt mixtures with 4% steel wool fibers were considered.

When heating with a single-turn coil, the steel slag coarse aggregate specimens showed similar surface and depth temperature gradients, regardless of induction frequency. However, for asphalt mixtures with porphyry coarse aggregate, the temperature gradient decreased with increasing frequency.

With a double-turn coil, the surface temperature gradient for asphalt mixtures with steel slag aggregate remained nearly constant across frequencies, while the depth gradient increased by about 10 °C as frequency increased. In contrast, for porphyry coarse aggregate, both surface and depth gradients decreased with frequency, resulting in the highest heating homogeneity observed in this study for the mixture with porphyry coarse aggregate and 4% fiber content.

Finally, with a centered two-turn coil, more heterogeneous heating was observed for asphalt mixtures with porphyry coarse aggregate, with greater surface gradients as the frequency increased. No significant differences in temperature gradients were noted for mixtures with steel slag aggregate.

Thus, it can be concluded that the frequency does not significantly affect heating homogeneity in asphalt mixtures with steel slag coarse aggregate. However, for mixtures with porphyry coarse aggregate, heating becomes more homogeneous with increasing frequency, especially in depth distribution. This behavior, contrary to expectations, suggests that asphalt mixtures do not behave purely as conductive materials, and assumptions based on conductive material behavior may lead to incorrect conclusions when studying self-healing asphalt.

Regarding coil shape, more homogeneous heating was achieved with the double-turn coil compared to the single-turn coil. However, when comparing the double-turn coil to the centered two-turn coil, better heat distribution was observed with the former. This is likely due to the accelerated surface heating with the centered two-turn coil, which generates higher temperatures near the coils and increases both the surface and depth temperature gradients, with a more pronounced effect at the surface. To prevent surface damage and ensure the internal bitumen flow, it is essential that heating is as homogeneous as possible, as high temperature gradients could harm the surface bitumen, limiting the healing effect.

**Figure 9:**
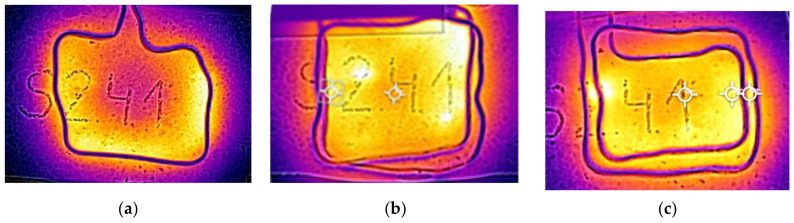
Influence of type of coil: (**a**) single-turn coil, (**b**) double-turn coil, and (**c**) two-turn centered coil.

Figure 9 shows the surface temperature distribution once 100 °C is reached, considering each type of coil.

## 4. Discussion

In the current study, the understanding of the induction heating process for asphalt mixtures is enhanced, focusing on the effects of current intensity, frequency, and coil shape. While extensive research has explored the self-healing properties of asphalt through induction heating [5], specific parameters influencing this heating process have been inadequately examined [17]. The findings from this research contribute to filling this gap by demonstrating that variations in current intensity, frequency, and coil design significantly affect the heating efficiency and distribution of temperature in different asphalt mixtures.

A particularly noteworthy observation is the superior heating performance of asphalt mixtures incorporating natural aggregates, specifically porphyry coarse aggregate, when compared to those formulated with steel slag aggregate. This finding challenges the conventional emphasis on steel slag, which has been widely used in prior studies [14,15,16]. The results indicate that porphyry coarse aggregate not only heats up more rapidly but also achieves a more homogeneous temperature distribution under controlled conditions. This advantage may be attributed to the physical and thermal properties of the porphyry aggregate, which could enhance the induction heating process. Such insights resonate with previous studies that suggest the choice of aggregate plays a pivotal role in determining the performance characteristics of asphalt mixtures [11]. Consequently, these results underscore the importance of considering the aggregate type when formulating asphalt mixtures intended for self-healing applications.

The optimization of induction heating parameters emerges as a critical factor for the successful implementation of this technology in practical applications. The results of this study demonstrate that adjusting the current intensity and frequency can lead to substantial improvements in heating efficiency, with direct implications for reducing both costs and time during maintenance and rehabilitation activities. Specifically, asphalt mixtures with porphyry aggregate not only heated faster but also achieved a more uniform temperature distribution, which is crucial for enhancing the self-healing process. While steel slag aggregates showed slightly lower heating efficiency, their use presents a sustainable alternative by recycling industrial waste materials, contributing to environmental sustainability. This aligns with findings from previous studies, such as Anupam et al. [5], who emphasized the need to optimize heating parameters to enhance the self-healing capabilities of asphalt, suggesting that such advancements could lead to improved pavement performance and reduced lifecycle costs.

Furthermore, in the context of low-carbon maintenance, the utilization of recycled materials with conductive properties is beneficial for the sustainability of asphalt pavements through thermally induced healing technologies [3]. There is also an urgent need to develop energy-efficient and cost-effective electromagnetic induction vehicles. These advances could significantly transform standard maintenance practices by reducing environmental impacts and enhancing the longevity and functionality of asphalt pavements.

However, this study is not without limitations. The investigation primarily focused on the heating characteristics of various asphalt mixtures under controlled laboratory conditions, which may not fully replicate the complexities encountered in real-world scenarios. For instance, environmental factors such as moisture, temperature fluctuations, and traffic loads could influence the induction heating process and the subsequent performance of the asphalt mixtures. Moreover, it is essential to confirm the observed behaviors regarding the heating characteristics of the mixtures with varying aggregate types and other parameters in further research. Our hypothesis suggests that the porosity inherent in steel slag aggregates may contribute to heat dissipation within their voids, potentially complicating their heating efficiency. This raises questions about the physical implications of such behavior, including whether these materials might struggle to maintain heat once it has been established.

Therefore, future research should extend this work by exploring the self-healing properties of these asphalt mixtures under diverse induction heating conditions. Such investigations are essential for validating the findings of this study and for understanding how different material compositions and environmental factors may impact the induction heating process and the resulting self-healing capabilities.

In summary, the findings of this study highlight the critical need for further exploration into the parameters governing induction heating in asphalt mixtures. A comprehensive understanding and optimization of these factors are essential for harnessing the full potential of self-healing asphalt technologies, to become available for their development and industrial application. This could ultimately lead to more sustainable and efficient road maintenance solutions, paving the way for enhanced infrastructure durability and performance.

## 5. Conclusions

In this study, the essential factors influencing the induction heating process in self-healing asphalt mixtures are successfully identified and characterized, emphasizing the roles of current intensity, frequency, ferromagnetic additive percentage, aggregate type, and coil design. The main findings of this study are summarized below:

- Current Intensity and Heating Rate: Increasing the current intensity significantly enhances the heating rate, with asphalt mixtures such as SHAC S4 and SHAC P4 reaching 100 °C in less than half the time at maximum current (I3) compared to minimum current (I1) settings.
- Coarse Aggregate Types: Asphalt mixtures containing porphyry aggregate exhibited faster heating rates and lower specific heat capacities, suggesting superior heating performance compared to those using steel slag aggregate. Notably, even though steel slag aggregates resulted in slightly lower heating efficiency, their incorporation offers a sustainable alternative by utilizing waste materials from the steel industry, thereby reducing the environmental impact associated with quarrying natural aggregates.
- Induction Frequency and Heating Distribution: While the frequency did not significantly impact the rate of surface heating, it played a crucial role in achieving a more homogeneous heating profile in porphyry aggregate specimens at higher frequencies. This uniform heating is critical for optimizing self-healing mechanisms, as it facilitates better bitumen flow within the matrix, enhancing its ability to fill cracks.
- Coil Design and Heating Uniformity: The double-turn coil demonstrated the most uniform heating distribution, promoting efficient self-healing by ensuring that all parts of the asphalt mixture reach optimal temperatures simultaneously, thus maximizing the healing potential.
- Sustainability and Self-Healing Potential: The incorporation of steel wool fibers not only reduces the energy required to achieve effective heating but also enhances the self-healing capabilities of asphalt pavements, increasing their resilience to wear and damage.
- Practical Implications: In this study, the importance of optimizing induction heating parameters for the practical application of self-healing asphalt technologies was highlighted. These optimized mixtures hold significant promise for reducing road maintenance costs, increasing infrastructure longevity, and minimizing the environmental impact associated with traditional asphalt production.
- Future Research: In this study, the need was emphasized for further exploration of self-healing asphalt mixtures, particularly regarding their performance in real-world scenarios. Key factors such as moisture and temperature variations must be considered in future investigations. Additionally, the impact of the porosity of materials like steel slag on heating efficiency warrants further study. Future research should focus on validating the self-healing capabilities of these mixtures under various induction heating conditions and assessing their long-term performance in practical applications.

In summary, the implementation of self-healing asphalt mixtures, particularly those using optimized parameters and sustainable materials, offers significant advantages for road maintenance, paving the way for more sustainable and efficient infrastructure solutions.

## Figures and Tables

**Table 1:** Particle size distribution of materials.

Type of Aggregate	Fraction	Test Sieves for Aggregates UNE-EN 933-2 [24] (mm)								Density (g/cm^3^)
		22	16	8	4	2	0.5	0.25	0.063	
Limestone filler	-	100	100	100	100	100	100	98	78	0.53
Limestone fine aggregate	0/4	100	100	100	99	71	26	13	0	2.712
Porphyry coarse aggregate	4/11	100	100	57	1	0	0	0	0	2.760
	12/18	100	61	1	1	1	1	1	1	2.735
Steel slag coarse aggregate	4/11	100	100	30	1	0	0	0	0	3.708
	10/20	100	69	2	1	1	1	0	0	3.737
AC16 surf S *	-	100	90–100	64–79	44–59	31–46	16–27	11–20	4–8	-

* Particle size and grading envelopes specifications (PG-3) [25].

**Table 3:** Bulk density of asphalt mixtures.

Type of Asphalt Mixture	Bulk Density (g/cm^3^)
SHAC S2	2.680
SHAC S4	2.705
SHAC P2	2.377
SHAC P4	2.390

## Data Availability

The raw data supporting the conclusions of this article will be made available by the authors on request.
